# Occupational exposure to asbestos and lung cancer in men: evidence from a population-based case-control study in eight Canadian provinces

**DOI:** 10.1186/1471-2407-12-595

**Published:** 2012-12-13

**Authors:** Paul J Villeneuve, Marie-Élise Parent, Shelley A Harris, Kenneth C Johnson

**Affiliations:** 1 Population Studies Division, Health Canada, Ottawa, Ontario, Canada; 2 Division of Occupational and Environmental Health, Dalla Lana School of Public Health, University of Toronto, Toronto, Canada; 3 Occupational Cancer Research Centre, Toronto, Ontario, Canada; 4 INRS-Institut Armand-Frappier, University of Quebec, Laval, Quebec, Canada; 5 Division of Epidemiology, Dalla Lana School of Public Health, University of Toronto, Toronto, Canada; 6 Prevention and Cancer Control, Cancer Care Ontario, Toronto, Ontario, Canada; 7 Health Promotion and Chronic Disease Prevention Branch, Public Health Agency of Canada, Ottawa, Canada

**Keywords:** Lung cancer, Asbestos, Cigarette smoking, Case–control, Occupational epidemiology

## Abstract

**Background:**

Asbestos is classified as a human carcinogen, and studies have consistently demonstrated that workplace exposure to it increases the risk of developing lung cancer. Few studies have evaluated risks in population-based settings where there is a greater variety in the types of occupations, and exposures.

**Methods:**

This was a population based case–control study with 1,681 incident cases of lung cancer, and 2,053 controls recruited from 8 Canadian provinces between 1994 and 1997. Self-reported questionnaires were used to elicit a lifetime occupational history, including general tasks, and information for other risk factors. Occupational hygienists, who were blinded to case–control status, assigned asbestos exposures to each job on the basis of (i) concentration (low, medium, high), (ii) frequency (<5%, 5-30%, and >30% of the time in a normal work week), and (iii) reliability (possible, probable, definite). Logistic regression was used to estimate odds ratios (ORs) and their corresponding 95% confidence intervals (CI).

**Results:**

Those occupationally exposed to (i) low, and (ii) medium or high concentrations of asbestos had ORs for lung cancer of 1.17 (95% CI=0.92 – 1.50) and 2.16 (95% CI=1.21-3.88), respectively, relative to those who were unexposed. Medium or high exposure to asbestos roughly doubled the risk for lung cancer across all three smoking pack-year categories. The joint relationship between smoking and asbestos was consistent with a multiplicative risk model.

**Conclusions:**

Our findings provide further evidence that exposure to asbestos has contributed to an increased risk of lung cancer in Canadian workplaces, and suggests that nearly 3% of lung cancers among Canadian men are caused by occupational exposure to asbestos.

## Background

Lung cancer continues to be the leading cause of cancer among Canadian men, and in 2012, it was estimated that 13,300 men would be diagnosed with lung cancer and 10,800 would die of it [1]. While cigarette smoking is recognized as the leading cause of lung cancer, many occupational exposures, including asbestos, have also been shown to increase risk. Asbestos is a term used to describe six naturally fibrous minerals, and one of these, chrysotile, accounts for 95% of the asbestos ever used worldwide, and until recently was the only type produced in Canada [2]. All forms of asbestos have long been recognized as human carcinogens by the United States Environmental Protection Agency [3], the International Agency for Research on Cancer [4], and the National Toxicology Program [5]. This conclusion is based largely on unequivocal evidence assembled from epidemiological studies that have found excesses of lung cancer and mesothelioma in highly exposed textile workers, miners, and cement factory workers [4,6].

Today, more than 90% of the asbestos produced worldwide is used to manufacture asbestos sheets and pipes [7]. The World Health Organization has estimated that approximately 125 million individuals continue to be exposed to asbestos in the workplace [8]. Occupational exposure to asbestos in Canada has decreased dramatically over the past two decades due to provincial occupational health and safety controls that have been implemented. While those involved in the mining of asbestos are at higher risk of developing asbestos-related disease, the precautions offered to these workers to limit exposure are greater than those unwittingly exposed through other trades. Overall, the mining of asbestos in Canada has decreased dramatically, and in 2011, for the first time in over 130 years, production was halted [9], Today, in Canada, the most common sources of asbestos exposure arise from the repair, renovation, and demolition of older (pre-1980) buildings.

Relatively few studies have examined associations between workplace exposure to asbestos and lung cancer using a population-based design. Population-based designs provide important features that include an ability to estimate risks over a wider range of exposure levels than those typically reported in industry-specific studies. They provide the opportunity to characterize the frequency and nature of exposures in the general population. Moreover, because such studies cover diverse occupational groups, there is a reduced impact of confounders that may be specific to particular occupations. Recently, a population-based case–control study in Montreal found that workers with substantive exposure to asbestos had a greater risk of lung cancer, however, this finding did not achieve statistical significance (odds ratio (OR) =1.78, 95% CI=0.94, 3.36) [10]. Cumulative exposure was positively associated with lung cancer risk in a case–control study in Stockholm, Sweden [11], while a multi-center European case–control study found no association between occupational exposure to asbestos and lung cancer in six Central and Eastern European countries, but a nearly twofold (OR=1.85, 95% CI=1.07-3.21) increased risk was observed among UK workers [12].

While both cigarette smoking and asbestos are recognized lung carcinogens, there remain uncertainties about how they operate together to increase the risk of lung cancer. Attempts to understand the joint effects of smoking and asbestos on the risk of lung cancer extend back to Selikoff et al.’s seminal work in the late 1960s [13]. A subsequent review of this literature suggested that the interactive effects are multiplicative [14], which implies that asbestos exposure increases the risk of lung cancer by the same factor in smokers and non-smokers alike. An additive relationship, on the other hand, would assume that the effects of asbestos exposure and smoking are independent. Other reviews [15,16] and a meta-analysis [17] have suggested that the combined effects of smoking and asbestos are more than additive but less than multiplicative. This conclusion is consistent with very recent work by Frost et al. that revealed interactions that were greater than additive, although the multiplicative association could not be rejected [18]. Apart from the studies by Gustavsson et al. and Pintos et al., we know of no other research that has evaluated the joint relationship between asbestos and smoking on lung cancer risk *in the general population* where exposure levels are much lower than in asbestos workers, yet with fewer precautions and protections offered to reduce exposure. In the Gustavsson et al. study, the association between asbestos and smoking on lung cancer risk was found to be between additivity and multiplicativity [11]. In the Montreal study, the association was found to be sub-multiplicative [10]. To add to this knowledge, we examined the joint relationship between smoking and asbestos in this population-based case–control study.

With this background, the primary objective of our study is to build upon past research by reporting on the association between occupational exposure to asbestos and lung cancer among Canadian men. The secondary objective of the study is to evaluate the combined effects occupational exposure to of asbestos and cigarette smoking on the risk of lung cancer.

## Methods

### Study population

A case–control study design was used to address the research objectives, and the data come from the lung cancer case–control component of the National Enhanced Cancer Surveillance System (NECSS). The overall objective of the NECSS was to improve our understanding of both environmental and occupational determinants of cancer [19]. The NECSS was a collaborative project between the Public Health Agency of Canada and cancer registries in eight Canadian provinces (British Columbia, Alberta,Saskatchewan, Manitoba, Ontario, Nova Scotia, Newfoundland, and Prince Edward Island). There were no subjects (cases or controls) from the province of Quebec. Detailed information was collected from cases and controls for a number of potential risk factors including: sociodemography, anthropometry, diet, smoking, exposure to second hand smoke, and participation in physical activities. Individuals were also asked to provide lifetime residential and occupational histories. Questionnaires were administered between 1994 and 1997.

The NECSS endeavoured to collect information for each incident cancer within three months of diagnosis. Among men, there were a total of 3,718 histologically confirmed lung cancer cases (ICD-9 rubric 162) identified between 1994 and 1997. Letters were sent to the physicians of 3,033 (81.6%) of these cases to solicit their participation. Physician consent was obtained and questionnaires were mailed to 2,548 (69%) of the cases; physician consent was refused for 229 (6%) of all eligible cases and 653 (18%) were deceased at the time of the request and therefore excluded. Completed questionnaires were returned by 1,736 of the 2,548 cases who were mailed a questionnaire yielding an overall response rate of 68.1%.

The NECSS assembled a series of controls from the general population. For 5 provinces, controls were identified through provincial health insurance plans (Prince Edward Island, Nova Scotia, Manitoba, Saskatchewan and British Columbia). These insurance plans cover more than 95% of residents in the province. Elsewhere, either random digit dialing (Newfoundland and Alberta), or property assessment data (Ontario) were used as the sampling frame to recruit controls. Frequency matching to the overall case grouping (19 types of cancers) was used to select controls with similar age and sex distribution, such that there would be at least one control for every case within each sex and 5-year age group for any specific cancer site within each province. In total, questionnaires were mailed to 4,270 men identified as possible controls in the 8 provinces. Approximately 7% of these (n=287) were returned because the address was incorrect, and no updated address could be found through publicly available sources. In all, 2,547 male controls returned completed questionnaires, representing 64% of those contacted and 60% of those ascertained.

For the purposes of our analyses, we restricted the study population to only include men given that we expected few women to have been exposed to asbestos in the workplace. We used the same analysis file previously used to evaluate associations between diesel engine exhaust emissions and lung cancer which excluded individuals under the age of 40, and those who had not worked for at least one year [20]. In the NECSS, among all participating incident lung cancer cases only 0.7% (n=13) were diagnosed before the age of 40; the corresponding number of controls excluded to meet the age requirement was 438. A total of 42 cases and 56 controls were excluded because their reported length of employment was less than one year. After applying these exclusion criteria we were left with a total of 1,681 cases and 2,053 controls.

### Occupational assignment of exposures

Cases and controls were asked to provide information for each job held in Canada for at least 12 months from the time they were 18 years old until the time of interview. Information sought for each job included: job title, main tasks, type of industry, location, and the start and end dates of employment. A total of 15,646 jobs were identified, of these 15,234 (97.4%) jobs contained sufficient information for exposure assessment. No exposures were assigned for jobs that were self-reported to be retirement (n=185), disability (n=10), and unemployment (n=8).

Occupations and industry titles were assigned by one of two hygienists, who were blinded to case–control status, using the Canadian Classification and Dictionary of Occupation codes (originally published in 1971 with revisions up until 1986), and Standard Industrial Codes [21]. The hygienist coded each job on the basis of exposure to known or suspected lung cancer carcinogens. These exposures included: asbestos, diesel and gasoline engine exhaust emissions, and crystalline silica. This assessment was guided by the scientific and technical literature, consultation with experts, and a review of existing databases of exposure assessment. The assignment of workplace exposures took into account the manner that asbestos was used over the years. For example, before 1976, drywall installers used dry-wall joint cement that contained asbestos, while after 1980 asbestos was banned in this cement.

The assignment of occupational exposures was done according to three dimensions: concentration, frequency and reliability. The frequency of exposure was assigned based on the proportion of work time during a normal work week that the subject was exposed; this assignment took into account whether the work was part-time or seasonal in nature. *‘Low’* frequency corresponded to less than 5% of the work time, *‘Medium’* between 5% and 30%, and *‘High’* represented more than 30%. Concentration was assessed on a relative scale. For each substance, benchmarks were established and exposures were coded with respect to these benchmarks. Non exposure was interpreted as exposure up to background levels found in the general environment. The relative benchmarks for concentration levels used by our team of hygienists were *‘Low’* for welders and boiler operators, *‘Medium’* for boiler and pipe insulators and marine firemen and *‘High’* for miners and insulation workers (blowers and sprayers). It is very difficult to provide a reliable estimate of the absolute number of fibres per unit of volume corresponding to the different exposure levels. However, as a crude indicator, we can suggest that our ‘Medium’ level corresponded roughly to the 1976 American Conference of Governmental Industrial Hygienists threshold limit values (TLV) given that these values were in force in Canada in 1983 at a time when our study subjects were working. Specifically, the TLV for chrysotile asbestos fibers over 5 microns was 5 fibres per/cc in these Quebec guidelines. Finally the third dimension of exposure, reliability, refers to the hygienists’ degree of confidence that the exposure was actually present in the job under evaluation; *‘Low’* refers to a possible exposure, *‘Medium’* to a probable exposure and *‘High’* to a certain exposure. Estimates of the inter-rater reliability of the exposure assignment method, which were based on the work of chemists from the group that conducted the exposure assessment our study, lend credibility to the validity of the approach we used. Specifically, Goldberg et al. reported that the percent agreement among raters was between 95% to 98% with a Cohen’s kappa from 0.5 to 0.7 [22].

### Statistical analysis

We constructed several metrics to characterize occupational exposure to asbestos. These metrics included: ever exposed, highest attained concentration (high, medium, low), as well as a duration of exposure. Given the small number of individuals that had high concentrations of exposure, we combined medium and high into one group. Those with a low reliability score (“possibly exposed”) were assumed to have had no exposure.

Logistic regression was used to estimate the odds ratios (OR) and their corresponding 95% confidence intervals (CI) for the various exposure metrics. Adjustments were made for the potential confounders: age, cigarette smoking, socioeconomic status, exposure to second hand smoke, and occupational exposure to silica, and diesel exhausts. Occupational exposure to silica, and diesel engine exhausts were assigned to the cases and controls using the same methodology that was used for asbestos. Silica and diesel exposures were modelled as cumulative time-weighted measures. While gasoline engine emission exposure measures were also derived for the cases and controls, they did not confound the risk estimates for asbestos, and therefore, were not included in the models as adjustment factors. Multivariable models were adjusted for cigarette smoking through the use of a pack-years variable which incorporated aspects of both smoking duration and intensity. Cigarette pack-years were defined as the number of years of smoking an average of 20 cigarettes per day. For exposure to second-hand smoke, a composite measure was used that took into account lifetime exposures received both at home, and in the workplace [23]. It was derived as a function of the number of years of exposure that incorporated both the number of regular smokers that lived in each residence, and the number of smokers who smoked regularly in the subjects’ immediate work environment

The joint effect of smoking and occupational exposure to asbestos was first examined by estimating the odds ratios for cross-classification categories of cigarette pack-years (<10, 10 - <40, ≥40) and the highest attained occupational exposure to asbestos (none, low, medium/high). The small numbers of lung cancers among never smokers (n=34; 2% of all cases) precluded a separate evaluation of asbestos risks in this group. The odds ratios and 95% confidence intervals were estimated for eight cross-classification categories, while the ninth category (no asbestos exposure, < 10 cigarette pack-years) was used as the referent. The joint effects of smoking and asbestos on lung cancer risk were evaluated using two previously derived indices: the *Synergy* (S) [24] and *Multiplicativity* (V) [25]. We followed a similar approach that Frost et al. used to evaluate the relationship between asbestos and smoking and lung cancer in workers in Great Britain [18]. We used our derived odds ratios (ORs) to calculate the index S [24] as follows:

$$S=\frac{OR_{\mathrm{AS}}-OR_{0}}{OR_{A}+OR_{S}-2OR_{0}}$$

Where OR_A_ is the odds ratio of lung cancer exposed to ‘medium or high’ levels of asbestos among those with little to no smoking history (<15 pack-years), OR_S_ is the odds ratio of lung cancer among smokers (≥ 40 pack-years) with no exposure to asbestos, OR_AS_ is the odds ratio of lung cancer among smokers (≥ 40 pack-years) exposed to asbestos, where each odds ratio is estimated relative to the referent group of men who had accrued less than 10 cigarette pack-years and were not exposed to asbestos (OR_0_).The Multiplicativity index was calculated as:

$$V=\frac{OR_{0}OR_{\mathrm{AS}}}{OR_{A}OR_{S}}$$

A value that exceeds one for the *S* index suggests an interactive effect between smoking and asbestos exposure on lung cancer that could imply a multiplicative effect. In contrast, a value of *S* near one suggests that the two risk factors would operate in an additive fashion on the risk of lung cancer. For the *V* index, a value of one indicates a multiplicative interaction, whereas as values greater and less than one indicate an interaction that is more or less than multiplicative, respectively.

### Ethics approval

The participating provincial cancer registries obtained approval of the NECSS study protocol through their respective ethics review boards. All participants provided informed consent.

## Results

Of the 15,234 occupations ever held by the study subjects, a total of 801 were coded as having either ‘probable’ or ‘definite’ exposure to asbestos. The most commonly reported exposed occupations were mechanics and repairmen, stationary engine and utility workers, pipefitters, and construction workers (Table 1). Water transport operating occupations represented the only group deemed to have a high frequency of exposure to asbestos. Specific jobs included in this group that worked on ships included: deck officers, engineering officers, deck crew, engine and boiler room crew workers.

**Table 1:** Most frequent occupations among the 801 jobs held by subjects that were classified as having probable or definite exposure to asbestos

	**SOC**	**Number of jobs**	**%**	**Most common exposure coding**^**A**^		
				**Confidence**	**Frequency**	**Concentration**
Mechanics and Repairmen (except electrical)	8580 – 8589	214	26.7	Probable	Low	Low
Stationary Engine and Utilities Equipment	9530 - 9539	124	15.0	Probable	Medium	Low
Pipefitting	8791	89	11.1	Probable	Low	Low
Construction	8733	79	9.9	Probable	Low	Low
Metal shaping occupations	8330 – 8339	48	6.0	Probable	Medium	Low
Fabricating, assembling electrical and electronics	8530 – 8539	34	4.2	Probable	Medium	Low
Water Transport Operating Occupations	9151 – 9159	42	5.2	Probable	High	Low
Firefighters	6111	31	3.9	Definite	Medium	Low
Plasterers	8784	23	2.9	Probable	Medium	Low
Total		801	84.5			

A – defined by highest percentage.

A total of 233 cases and 224 controls, respectively, were exposed to asbestos at some point during their lifetime occupational history (Table 2). Those who were ever exposed to asbestos had a 28% increased risk of lung cancer relative to those who were not (OR=1.28, 95% CI: 1.02, 1.61). The risks according to highest concentration of occupational exposure ever attained were more pronounced. Only two cases and one control reported working in a job with an assigned ‘high’ concentration of exposure. As a result, we combined ‘medium’ and ‘high’ concentrations into one category. Those who had ever been exposed to medium or high levels had a more than twofold increase in risk (OR=2.16, 95% CI=1.21-3.88).

**Table 2:** Adjusted odds ratios of lung cancer in relation to occupational exposure to asbestos

**Occupational exposure**	**Cases**	**Controls**	**Odds ratio**^**A**^**and 95% CI**		**Odds ratio**^**B**^**and 95% CI**	
Unexposed*	1448	1829	1.0	-	1.0	-
Ever exposed	233	224	1.31	1.07 – 1.59	1.28	1.02 – 1.61
Highest attained exposure						
Unexposed	1448	1829	1.0	-	1.0	
Low	194	200	1.22	0.99 – 1.51	1.17	0.92 – 1.50
Medium / High	39	24	2.02	1.20 – 3.97	2.16	1.21 – 3.88
Total	1681	2053				
Duration of exposure (years)						
< 10	88	68	1.68	1.21 – 2.33	1.60	1.10 – 2.33
10 - < 20	46	50	1.08	0.74 – 1.69	0.89	0.56 – 1.42
≥ 20	87	103	1.05	0.78 – 1.42	1.18	0.84 – 1.66

A – Adjusted for age, province,

B – Adjusted for age, province, cigarette pack years, occupational exposure to diesel and silica, exposure to second hand smoke.

**Figure 1:**
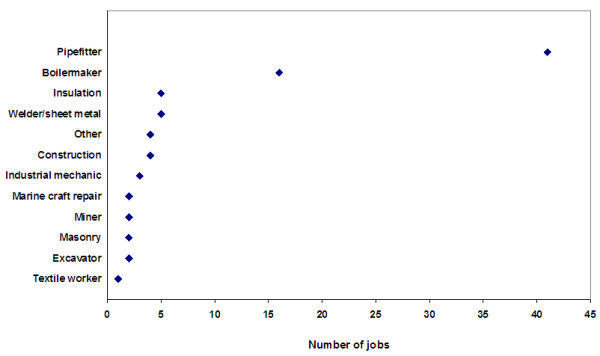
Most common occupations among mean with medium or high concentration levels of asbestos, NECSS lung cancer case-control study.

We found that duration of occupational exposure to asbestos was not related to the risk of lung cancer (Table 2). When we modeled duration of exposure as a continuous variable, the adjusted odds ratio of lung cancer for an increase in 10 years of exposure was 1.03 (95%% CI=0.94-1.13). This risk increased to 1.13 (95% CI=0.84-1.52) when analyses were restricted to those who were only exposed to medium or high concentrations; this result however was not statistically significant (p=0.44). The frequency of the jobs that were deemed to have ‘medium’ or ‘high’ concentrations of asbestos is presented in Figure 1. The most common of these jobs were pipefitters and boilermakers, and insulators.

None of the first-order interaction terms between cigarette smoking pack-years and the three measures of asbestos exposure were statistically significant. The corresponding p-values for the smoking interaction terms with ‘ever’, ‘highest attained’ and ‘duration’ asbestos exposure were 0.33, 0.77, and 0.88, respectively.

Stratified analyses of highest attained asbestos exposure across cigarette pack years categories are presented in Table 3. There was an approximate two-fold increase in risk among those with ‘medium’ or ‘high’ occupational exposure to asbestos relative to those with no such exposure in each of the three pack-year categories. This is consistent with a multiplicative relationship between the two factors. Those who had at least 40 pack-years of smoking and were exposed to medium or high asbestos levels had the highest risk of lung cancer; relative to those with no asbestos exposure, and less than 10 cigarette pack-years, their risk nearly 38-fold higher (OR=38.59, 95% CI=10.78-138.08) (Table 4). The calculated values of the S and V indices were 2.10 and 0.99 respectively, supporting the notion that the interaction between asbestos and smoking is multiplicative.

**Table 3:** Adjusted odds ratios* and 95% C.I. according highest occupational exposure to asbestos across cigarette pack-year smoking categories

	**Cigarette smoking (pack-years)**								
**Highest occupational exposure to asbestos**	**< 10**			**10 - <40**			**≥ 40**		
	N	OR*	95% C.I.	N	OR*	95% C.I.	N	OR*	95% C.I.
None	84	1.0	-	630	1.0	-	678	1.0	-
Low	13	1.54	0.79 – 3.00	90	1.28	0.91 – 1.80	85	0.88	0.58 – 1.35
Medium or high	2	2.01	0.39 – 10.43	20	2.30	1.09 – 4.84	16	2.50	0.73 – 9.28

N = number of lung cancer cases.

* adjusted for age, province, occupational exposure to diesel and silica, and second hand cigarette smoke.

**Table 4:** Synergy and multiplicative indices between asbestos exposure and cigarette smoking

**Cigarette smoking (pack-years)**	**Asbestos exposure**	**Label**	**Cases**	**Controls**	Odds ratio*	**95% CI**
< 10	None	R_0_	84	745	1.0	-
	Low	—	13	69	1.47	(0.77 – 2.81)
	Medium/High	R_A_	2	7	2.20	0.42 – 11.41)
10 - < 40	None	—	630	778	5.28	(3.90 – 7.14)
	Low	—	90	90	6.67	(4.41 – 10.10)
	Medium/High	—	20	13	10.39	(4.83 – 22.36)
≥ 40	None	R_S_	678	266	17.68	(12.90 – 24.22)
	Low	---	85	40	15.62	(9.72 – 25.09)
	Medium/High	R_AS_	16	3	38.59	(10.78 – 138.08)
Synergy Index					2.10	
Multiplicativity Index					0.99	

* adjusted for age, province, occupational exposure to diesel and silica, and second hand cigarette smoke.

## Discussion

This population-based study of men employed across a diverse range of jobs found that workplace exposure to asbestos was associated with an increased risk of lung cancer. This association persisted after adjusting for cigarette smoking, second hand smoke, and other occupational exposures previously implicated as possible risk factors for lung cancer. The approximate 28% increased risk observed among men ever exposed to asbestos is similar to the finding of Pintos et al. [10]. In their Montreal based case–control study, those who were exposed to asbestos had an odds ratio of 1.21, (95% CI=0.98-1.49) relative to those with no exposures. The population attributable risk (PAR) percent is often used to provide an estimate of the percentage of cases that be avoided if the putative exposure was eliminated [26] . We calculated the PAR in our study using the odds ratio of 1.28 among ever exposed, and an estimated prevalence of exposure of 11.3% (based on our control series). This yielded a PAR of 3.1% which suggests that a relatively small percentage of Canadian male lung cancer cases are due to occupational exposure to asbestos. Based on an estimated 13,300 incident lung cancers among men in Canada in 2012 [1] this would account for approximately 412 incident cases.

Our study provided support for a dose–response relationship between asbestos exposure and lung cancer as higher risks were observed among those who were ever exposed to ‘medium’ or ‘high’ concentrations of asbestos. Pipefitters accounted for nearly half of these cases and controls (41 of 87). While the limited number of subjects did not allow us to characterize risks for specific types of jobs, our results are consistent with a previously published study of Ontario pipe trade workers [27]. They reported a 53% increased risk of lung cancer mortality among pipefitters who had been registered trade members for at least 30 years, relative to the Ontario general population. However, their study was somewhat limited due to a lack of data on smoking. Our findings support the hypothesis that asbestos and cigarette smoking affect the risk of lung cancer in a multiplicative fashion.

In many occupational studies, duration of exposure is regarded as valid surrogate measure of cumulative exposure due to the inherent difficulties in retrospective studies to precisely characterize exposure intensity. In their Montreal case–control study, Pintos et al. found a higher risk of lung cancer among those exposed to asbestos for at least 20 years when compared to those exposed for shorter durations [10]. Duration of exposure was also positively associated with lung cancer risk in other industry-specific cohorts [28]. In contrast, we found that only intensity but not duration of exposure was associated with statistically significant increased risks of lung cancer. This observation is consistent with recently published findings on a cohort of workers employed in an asbestos reprocessing plant in the Calvados region of France [29]. In this study, Clin and colleagues observed that the average exposure to asbestos expressed in terms of fibers per ml was associated with pleuro-peritoneal mesothelioma, lung cancer, and colorectal cancer (p<0.05), however, no statistically significant associations were evident with duration of exposure for any of these three cancer sites. Other studies of asbestos workers have also found associations with intensity but not duration of exposure [12,30,31]. Our finding of a stronger positive association between duration of exposure at medium or high levels of asbestos when compared to durations spent at lower levels suggests that time exposed above a threshold level may be a relevant marker of risk. However, this finding should be interpreted cautiously as it based on a very small number of subjects who were exposed to either medium or high intensities.

It is well recognized that there is a lengthy latency period between the time of first exposure to an environmental carcinogen and the development of a solid tumour such as lung cancer. For example, the latency period associated with cigarette smoking and lung cancer has been estimated to be several decades following the initiation of smoking [32]. By extension, the increased risks of lung cancer due to exposure to asbestos observed in this study are a reflection of workplace exposures many years if not decades earlier. Indeed, among those classified has having ‘medium’ or ‘high’ concentrations to asbestos in the workplace, the start date of employment was after 1980 in only 6% of these jobs.

Participants in our study were asked to provide information for only those jobs that were held for at least one year. The exclusion of these short-term jobs raises the possibility that some exposure misclassification has been introduced. Previous analysis of 27.5 million workers found increased risks of lung cancer among those exposed to high levels of asbestos (20 to 40 fibers per cubic centimeter of air) for only a few months [33]. Under a classical error model where the possible exposure misclassification error arising from excluding these short term jobs is non-differential to case–control status, our risk estimates would be understated.

An important strength of this study was the availability of other risk factor data obtained through both the questionnaire, as well as expert-based coding of occupational histories. Unlike many other occupational case–control studies, we had extensive data on cigarette smoking, most notably, exposure to second hand smoke. This measure allowed our risk estimates to take into account lifetime exposure to second-hand smoke incurred at both home and workplace settings. In addition, the industrial hygienists also coded each job for possible exposure to other known or suspected lung carcinogens including: crystalline silica, gasoline and engine emissions. We recently found that occupational exposure to diesel but not gasoline engine emissions increased the risk of lung cancer; the risk of lung cancer was also increased among individuals exposed to crystalline silica [34]. The addition of these two covariates (diesel and silica) strengthened the association for asbestos by approximately 20%.

Approximately 68% of eligible cases and 64% of eligible controls completed a questionnaire. This raises the potential to introduce some bias in our risk estimates, and our results should be interpreted cautiously because of this possibility. However, for several reasons, we do not believe this bias fundamentally changes our results. First, observed associations with known and suspected risk factors such as cigarette smoking, and exposure to second-hand smoke are similar in direction and magnitude to risk estimates reported in other epidemiological studies. Moreover, our published findings for other occupational exposures within the same study population [34] are also consistent with the epidemiological literature. Lastly, the distribution of lung cancers by histology in our study is remarkably similar to population-based figures for North America [35] and provides some support for the generalizeability of these results to incident lung cancers in Canada. Unfortunately, the NECSS did not collect data from those diagnosed with mesothelioma, and therefore, we were unable to investigate associations with this endpoint.

We were unable to distinguish asbestos on the basis of fiber type. Asbestos fibers can be described according to two broad classes *serpentines* (phyllosilicates) and *amphiboles* (inosilicates) that differ substantially with respect to biopersistence and physical and chemical properties. Serpentines include chrysotile asbestos which is the predominant type of asbestos in Canada. The International Agency for Research on Cancer has determined that there is sufficient evidence to conclude that all these forms of asbestos can cause cancer in humans [4,6]. There remains considerable uncertainty regarding differences in lung cancer risk resulting from exposure to different types of asbestos fibers. A review of cohort studies where quantitative measurements of asbestos exposure were available demonstrated clearer and consistent associations between exposure and lung cancer for crocidolite or amosite [36]. On the other hand, associations from cohorts exposed primarily to crysotile asbestos were less consistent [37,38]. It is generally accepted that amphibole fibers are more harmful than chrysotile fibers for mesothelioma [36,39]. However, it has been argued that these differences are not all that important given that chrysotile is the most commonly used type of asbestos [40,41]. In our study, those who were determined to have been exposed to asbestos were believed to have been exposed to chrysotile, however, it is possible that some exposure to less prevalent yet more potent types of fibers occurred and was unaccounted for.

Another limitation of our study was the relatively small number of study subjects who were ever exposed to medium or high levels of asbestos. In total, there were only 39 cases and 24 controls exposed at these levels. These small numbers hindered our ability to characterize the joint relationship between smoking and asbestos exposure on the risk of lung cancer. It also limited our examination of the risks of lung cancer with exposure to asbestos according to different histological subtypes. Several studies have found associations that were most pronounced for adenocarcinoma subtypes [28,42-44], however, others did not [45-47]. The three most common histological types of lung cancer in our study population were squamous cell carcinoma (35%), adenocarcinoma (28%), and small cell carcinoma (15.9%) [34]. When we restricted analysis to adenocarcinoma, the odds ratio among those exposed to medium or high levels of asbestos increased from 2.16 to 3.14 (95% CI=1.50 – 6.58). However, the latter estimate was based on only 13 incident cases and therefore, our study has very limited statistical power to make inferences by histological type.

## Conclusions

In summary, the findings from this Canadian case–control study are consistent with the determination by international agencies that asbestos is a human lung carcinogen. While chrysotile asbestos is the predominant type of asbestos in Canada, it is possible that some of the workers in our study were exposed to other types of asbestos fibers. For this reason, and given the relatively small number of individuals exposed to medium and high exposure where the excess risks of lung cancer were found, we cannot conclusively attribute increased lung cancer risks to chrysotile. Despite the limitation, our findings provide further support that exposure to asbestos has contributed to an increased risk of lung cancer in Canadian workplaces.

## Abbreviations

CI: Confidence interval; OR: Odds ratio; PAR: Population attributable risk; NECSS: National enhanced cancer surveillance system; TLV: Threshold limit value.

## Competing interests

The authors have no competing interests to declare.

## Authors’ contributions

PV contributed to the design of the study, conducted analysis of the data, and took the lead in preparing the manuscript. SH contributed to the design of the study, and assisted in the development of the manuscript. KJ is the principle investigator of the NECSS and oversaw the original collection of the data, design of the questionnaire, and contributed to the writing of this manuscript. MEP oversaw the assignment of the occupational exposures for this study, contributed to the design, and played a prominent role in the writing of this manuscript. All authors read and approved the final manuscript.

## Authors’ information

The Canadian Cancer Registries Epidemiology Research Group comprised a principal investigator from each of the provincial cancer registries involved in the National Enhanced Cancer Surveillance System: Bertha Paulse, Newfoundland Cancer Foundation; Ron Dewar, Nova Scotia Cancer Registry; Dagny Dryer, Prince Edward Island Cancer Registry; Nancy Kreiger, Cancer Care Ontario; Erich Kliewer, Cancer Care Manitoba; Diane Robson, Saskatchewan Cancer Foundation; Shirley Fincham, Division of Epidemiology, Prevention and Screening, Alberta Cancer Board; and Nhu Le, British Columbia Cancer Agency.

## Pre-publication history

The pre-publication history for this paper can be accessed here:

http://www.biomedcentral.com/1471-2407/12/595/prepub
